# Cervical cancer heterogeneity: a constant battle against viruses and drugs

**DOI:** 10.1186/s40364-022-00428-7

**Published:** 2022-11-17

**Authors:** Qian Sun, Liangliang Wang, Cong Zhang, Zhenya Hong, Zhiqiang Han

**Affiliations:** 1 Department of Obstetrics and Gynecology, Tongji Hospital, Tongji Medical College Huazhong University of Science and Technology, Wuhan 430030, Hubei, China; 2 Department of Hematology, Tongji Hospital, Tongji Medical College, Huazhong University of Science and Technology, Wuhan 430030, Hubei, China

**Keywords:** Human papillomavirus, Tumor heterogeneity, Tumor microenvironment, Drug resistance, Immunotherapy

## Abstract

Cervical cancer is the first identified human papillomavirus (HPV) associated cancer and the most promising malignancy to be eliminated. However, the ever-changing virus subtypes and acquired multiple drug resistance continue to induce failure of tumor prevention and treatment. The exploration of cervical cancer heterogeneity is the crucial way to achieve effective prevention and precise treatment. Tumor heterogeneity exists in various aspects including the immune clearance of viruses, tumorigenesis, neoplasm recurrence, metastasis and drug resistance. Tumor development and drug resistance are often driven by potential gene amplification and deletion, not only somatic genomic alterations, but also copy number amplifications, histone modification and DNA methylation. Genomic rearrangements may occur by selection effects from chemotherapy or radiotherapy which exhibits genetic intra-tumor heterogeneity in advanced cervical cancers. The combined application of cervical cancer therapeutic vaccine and immune checkpoint inhibitors has become an effective strategy to address the heterogeneity of treatment. In this review, we will integrate classic and recently updated epidemiological data on vaccination rates, screening rates, incidence and mortality of cervical cancer patients worldwide aiming to understand the current situation of disease prevention and control and identify the direction of urgent efforts. Additionally, we will focus on the tumor environment to summarize the conditions of immune clearance and gene integration after different HPV infections and to explore the genomic factors of tumor heterogeneity. Finally, we will make a thorough inquiry into completed and ongoing phase III clinical trials in cervical cancer and summarize molecular mechanisms of drug resistance among chemotherapy, radiotherapy, biotherapy, and immunotherapy.

## Introduction

Human papillomavirus (HPV) causes an overwhelming majority of cervical cancers (CCs) and an alarmingly increased proportion of oropharyngeal cancers (OPCs). As the earliest discovered HPV-associated cancer, the tumorigenesis and infiltration of cervical cancer are closely relevant to the persistent infection and genome integration of HPV [1, 2].

Although with clear etiology, tumor heterogeneity still exists and gradually becomes a new challenge in the field of HPV-associated cancer research. Three concepts of heterogeneity need to be clarified: inter-patient heterogeneity, inter-tumoral heterogeneity, and intra-tumoral heterogeneity. Differences in tumor phenotypes and genotypes among individuals or distinct tumor sites are defined as inter-patient heterogeneity and inter-tumor heterogeneity respectively. In contrast, intra-tumoral heterogeneity means genomic, transcriptomic, epigenetic, or phenotypic differences within the same tumor lesion which are associated with therapeutic resistance and considerably more challenging [3]. Mendelian law of inheritance suggests that the free combination of genes is an important reason for the emergence of biodiversity [4]. As these probabilistic events encounter Darwinian adaptational selection over time, tumor cells and normal cells will continue to compete in different quadrants of time and space [5]. Genetic intra-tumoral heterogeneity, inter-tumoral heterogeneity, and inter-patient heterogeneity are reflected in a dynamic process of tumorigenesis, invasion, metastasis, or drug resistance [6–8]. To overcome the dilemma of precision therapy, it is necessary to break through each of these aspects. The rapid development of sequencing technology provides a platform for revealing tumor heterogeneity. Scanty knowledge has been uncovered on how heterogeneity plays roles in tumor pathogenesis and precision therapy until application of single-cell transcriptome analysis. The advances in single-cell RNA sequencing (scRNA-seq) include distinguishing neoplastic from normal tissue in individual patients and different disease states [9, 10].

In this review, we explore the heterogeneity of cervical cancers from the perspectives of HPV-induced tumorigenesis, internal changes of human genome and molecular mechanisms of drug resistance. The molecular and clinical features of cervical squamous cell carcinoma are discussed in major. In addition, cancer stem cells, cervical adenocarcinoma, and neuroendocrine carcinoma are described respectively in the last chapter. Firstly, we emphasize the significant contribution of alterations of genetic material and HPV gene integration differences in tumorigenesis. Furthermore, we summarize the mechanisms of intra-tumoral and inter-tumoral heterogeneity among inchoate and advanced cancers. Finally, we attempt to explain the huge differences in resistance to therapies among populations through tumor heterogeneity and provide feasible strategies for precise treatment.

## Inferring heterogeneity with HPV

### Heterogeneity of geographical distribution

In the 1980s, German pathologist Dr Hausen identified HPV as the explicit cause of cervical cancer which opened a new revolution in the etiology treatment of cancer. Moreover, HPV infection is also the cause of multiple cancers in both women and men, including anogenital cancer (anal, vaginal, vulvar, and penial) or head and neck cancer (oropharynx, oral and laryngeal) [11]. Recent studies indicate that over 90% of cervical and anal cancers, over 70% of oropharynx cancers, about 70% of vulvar and vaginal cancers, together with more than 60% of penile cancers are related to HPV [12, 13].

**Fig. 1:**
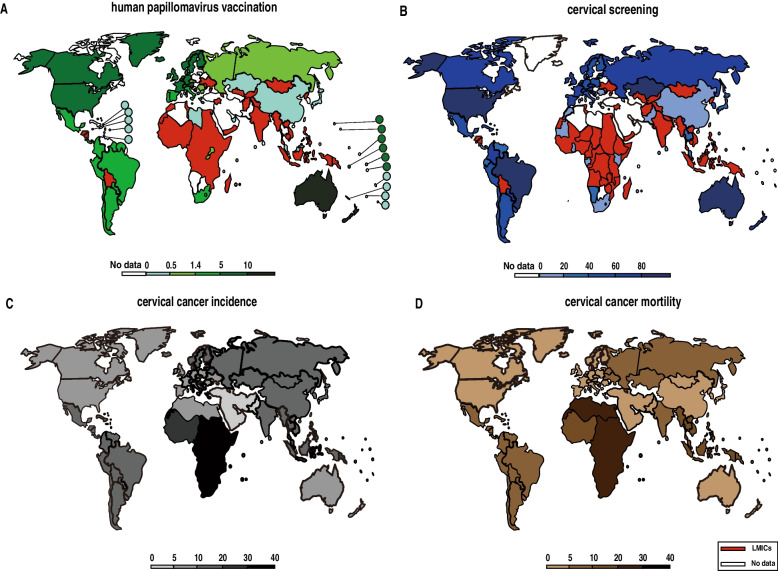
Global map of epidemiological data for tertiary prevention of cervical cancer. **A** vaccination coverage rates and **B** cervical cancer screening rates by country in 2014. **C** incidence and **D** mortality age-standardized rates per 100,000 by region in 2020. The full-course coverage data among the total female population are illustrated. 78 low-income and lower-middle-income countries involved in the WHO cervical cancer elimination project are highlighted in red. Source: GLOBOCAN 2020

Cervical cancer is the fourth most common cancer in women with 604,127 new cases and 341,831 deaths occurring worldwide in 2020 [14, 15]. The incidence and mortality have shown an obvious geographical imbalance between low-income and middle-income countries (LMICs) with high-income countries in cervical cancer patients. In LMICs, CC is the second most common cancer with an incidence rate of 18.8 per 100 000 women and a mortality rate of 12.4 per 100 000 women. In contrast, as a result of the availability of HPV prophylactic vaccines and standardized screening strategies, the incidence (11.3/100 000 women) and mortality (5.2/100000 women) of cervical cancer have decreased in high-income countries [14]. Vaccination and screening are effective in preventing cervical cancer, but they will impose a huge global economic burden. A systematic review has demonstrated that 106 million women have received at least one dose of HPV vaccine worldwide till 2014, but the HPV vaccination and standardized screening coverage in LMICs are still obviously low [16, 17].The world health organization (WHO) made a call for global action toward CC elimination in 2018, through vaccinating 90% of all girls under the age of fifteen, screening 70% of women at the age of 25, and treating 90% of precancerous lesions. The prediction simulation using the WHO Cervical Cancer Elimination Modelling Consortium (CCEMC) shows that the premature mortality rate of CC in 78 LMICs could be reduced by a third in the next 10 years. The WHO triple-intervention strategy would result in a 96.2% reduction by 2070, and 98.6% reduction by 2120. Famously, vaccination alone could reduce the mortality by 62.7% till 2070 and 89.5% till 2120. It is believed that with concerted global efforts, the incidence of cervical cancer in LMICs will be steadily reduced (Fig. 1) [18]. In 2019, the first domestic bivalent HPV vaccine was released and contributed to the HPV vaccination program in China [19]. This geographical distribution heterogeneity is therefore bound to become uniform gradually with the improvement of the global economic level and the implementation of prevention strategies.

### Heterogeneity of HPV infection types

**Fig. 2:**
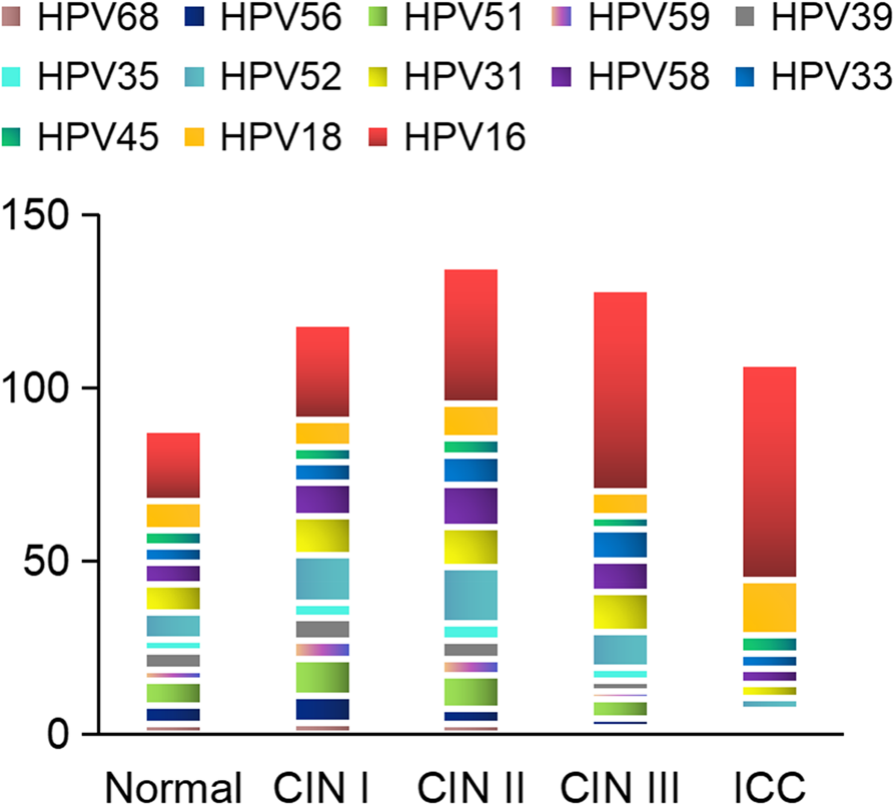
Histogram chart of HR-HPV infection distributions by different disease states. Normal: disease-free state; CIN: cervical intraepithelial neoplasia; ICC: invasive cervical cancer

The infection rate besides the infection site of different HPV types is heterogeneous across populations. Fifteen high-risk HPV (HR-HPV) types have been confirmed as carcinogenic viruses, as follows, 16,18, 31, 33, 35, 39, 45, 51, 52, 56, 58, 59, 68, 73, and 82. Among them, the cumulative infection rate of HPV16 and 18 accounts for 79% of the squamous-cell carcinomas, and accounts for 95% of the squamous-cell carcinomas together with HPV45, 31, 33, 52, 58, and 35 [20, 21]. A meta-analysis collated data from 115,789 HPV-positive patients has been performed to analyze the distribution of HR-HPV. The percentage of 13 HR-HPV infection distributions under different disease states are demonstrated in Fig. 2, and include HPV16, 18, 31,33, 35, 39, 45, 51, 52, 56, 58, 59 and 68. Among them, HPV16, 18 and 45 infections dominate in invasive cervical cancer( ICC) (ICC: normal ratios 3.1, 1.9 and 1.1, respectively) [22].

Another systematic meta-analysis collated data from 19 883 HIV-positive patients has been performed and analyzed the distribution of HR-HPV in 2017. Similarly, the ICC: normal ratios of HPV infections are 3.7 (HPV16), 2.5 (HPV18), and 2.6 (HPV45) respectively which is consistent with the conclusion in HIV-uninfected populations [23]. It is illustrated that HPV16, 18 and 45 positivity increase distinctly from normal cytology through squamous intraepithelial lesions to invasive cervical cancer which suggests that we should pay special attention to these types in cervical cancer screening.

On the other hand, in a large sample of healthy people screening, there is data to support a shift in the pre- and post-vaccine prevalence profile. HPV16, 18, 31, 52 and 58 were the most five common infection types in women with normal cytology in the pre-vaccine era [24]. However, infection rates of HPV52, 58, and 56 are increasing in the post-vaccine era [25, 26]. Whether bivalent and quadrivalent vaccines can provide cross-protection is controversial, but there is no doubt that the spectrum of HPV-associated squamous intraepithelial lesions and invasive cervical cancer will continue changing with the introduction of the 9-valent vaccine or even the 11-valent vaccine. Although cervical cancer is being treated earlier and earlier, it is still a constant battle against the ever-changing virus types.

### Heterogeneity of anti-viral immunity

**Fig. 3:**
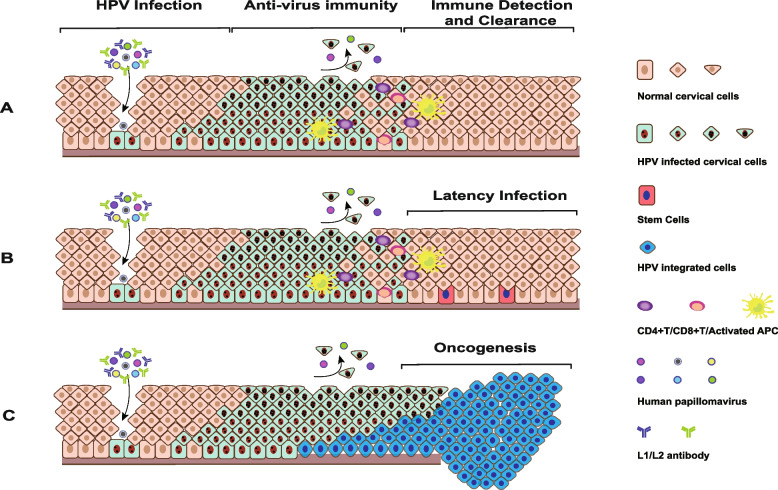
Clinical outcomes of different anti-viral immune states after HPV infection. **A** HPVs are completely eliminated by the body’s immunity; **B** A majority of HPVs is eliminated, a small percentage of latent basal layer stem cells still exist; **C** HPVs induce immunosuppression, gene integration, CIN and carcinogenesis

Upon HPV infection, the host cell immediately activates the innate and adaptive immunity to eliminate the virus [27]. Tumor heterogeneity of cervical cancer is reflected in the outcome of the battle between our immune system and virus invasion in the post infection microenvironment (PIM). HPV is undoubtedly one of the most important external factors mediating the heterogeneity of tumor development. HR-HPV type, duration of infection, virulence, human genomic instability and immune clearance will affect the tumorigenesis and development of carcinoma [28]. There are three outcomes of the battle between our immune system and HPV infection. Firstly, the virus is thoroughly cleared by our immune system. Secondly, the overwhelming majority of the virus is cleared, and only minority viruses that lie dormant can escape immunological recognition. Thirdly, the virus escapes immune recognition and integrates into the human genome, resulting in persistent infections and tumorigenesis [29, 30]. Fortunately, persistent high-risk HPV (HR-HPV) infection combined with oncogene genomic integration might lead development of normal cervical cells into intraepithelial neoplasia (CIN) or ICC in decades (Fig. 3).

PIM has been recognized as a complex and dynamic position with a collection of highly heterogenous cellular or molecular compounds, especially induced by the interaction between HPV-infected keratinocytes and immune cells. Specific cellular immune reactions and break down of immunosuppressive status are essential for effective virus clearance. Insufficient trafficking or maturation of Langerhans cells may lead to antigen-presenting disorder and CD8^+^ cytotoxic T lymphocyte (CTL) response impairment [31, 32]. Otherwise, the expression of MHC-I on the surface of keratinocytes have been down-regulated after HPV infection and recognition of CTLs will be avoided [33]. Except for CTLs, CD4^+^ T cells are essential in HPV clearance. An imbalance in T-helper 1(Th1) and Th2-type CD4^+^ T cells might be associated with immune dysregulation. Furthermore, the malfunction of NK cells is associated with immunosuppression [34].

### Heterogeneity of genomic instability and HPV integration

Key characteristics of PIM include immunosuppressive state, oxidative stress response, extracellular matrix (ECM) remodeling, and metabolic reprogramming [35]. Oxidative stress could amplify inflammatory responses and result in accumulation of DNA damage, mutations or genome instability [36]. Expression of matrix metalloproteases is also increased and associated with ECM remodeling and precancerous lesion occurrence [37]. Once the immune microenvironment remodeling that promotes the persistence of HPV infection is established, genomic integration and cytopathic changes occur continuously.

As the overexpression of oncoproteins E6 and E7 in the HPV-infected keratinocytes, E6 disrupts p53 degradation and alteration of cell regulation, on the other hand, E7 induces retinoblastoma (pRb) degradation and promotes cell proliferation [38]. E6 and E7 may also induce genomic instability and lead to carcinogenesis by abrogating cell-cycle checkpoints [39]. Growing evidence suggests that chromosomal instability is also a driving force for the oncogenic transformation of cervical cancer. High chromosomal instability Hela cells exhibit a higher karyotype heterogeneity and are related to KRAS signaling regulation [40].

**Fig. 4:**
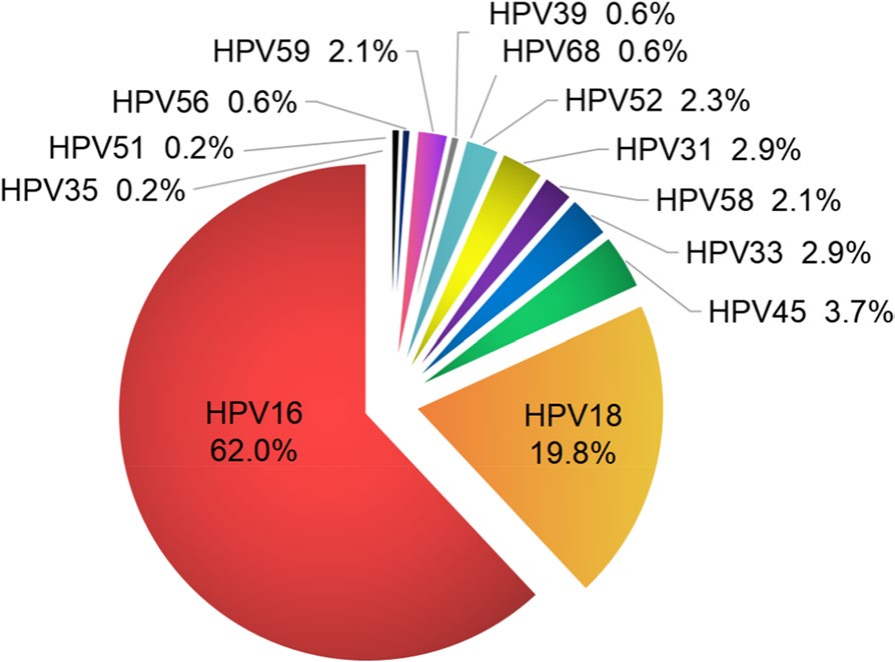
Pie chart of proportional distribution of reported HPV integration events by HR-HPV types. Only integration events detected through NGS or WGS data from human cervical cancer specimens are included (references: [39–43] and [44])

HPV is a small double-stranded DNA virus whose DNA fragments have the ability to integrate into the human genome. Associations between HPV integration and adjacent host genomic structural variation have been confirmed in HPV-positive cervical cancer cell lines. HPV16 integration has been detected firstly on chromosome 13q22 in SiHa cell lines in 1987 [41]. HPV 16, 18, and 33 viral integration has been detected in cervical squamous cell carcinomas by scientists as early as 1991 [42]. All integration events of the 13 HR-HPV subtypes have already been observed, and an unbalanced distribution of HR-HPV genotypes in cervical cancer has been detected. We have summarized six high-quality studies with HPV integration data of cervical cancer patients through next-generation sequencing (NGS) or whole-genome sequencing (WGS), and the proportion of integration events among different subtypes is analyzed. We can see that the integration of type 16 and 18 accounts for more than 80% of all samples. Other HR-HPVs are HPV45, 31, 33, 52, 58, 59, 39, 56, 68, 35 and 51 in a descending order of integration ratio (Fig. 4) [43–48]. We have summarized the high-frequency (more than 4 reported) disrupted genes by HPV integration and listed the hotspots, such as 3q28, 8q24, and 13q22. The top five reported genes are MACROD2, FHIT, POU5F1B, LRP1B and RAD51B (Table 1).

**Table 1:** Summary of high-frequency disrupted genes by HPV integration in cervical cancer

Gene	Integrations reported	Official full name	Gene ID	Location	Reference
MACROD2	13	Mono-ADP ribosylhydrolase 2	140733	20p12.1	[39–44]
FHIT	11	Fragile histidine triad diadenosine triphosphatase	2272	3p14.2	[39, 41, 42]
POU5F1B	11	POU class 5 homeobox 1B	5462	8q24.21	[41–43]
LRP1B	10	LDL receptor related protein 1B	53353	2q22.1	[39, 42]
RAD51B	10	RAD51 paralog B	5890	14q24.1	[39, 40, 44]
KLF12	9	Kruppel like factor 12	11278	13q22.1	[41, 42]
KLF5	9	Kruppel like factor 5	688	13q22.1	[39, 41, 42]
HMGA2	7	High mobility group AT-hook 2	8091	12q14.3	[42]
ERBB2	7	Erb-b2 receptor tyrosine kinase 2	2064	17q21.31	[40, 41, 44]
DMD	7	Dystrophin	1756	Xp21.2-p21.1	[39, 42, 44]
MAPK10	6	Mitogen-activated protein kinase 10	5602	4q21.3	[39, 42, 43]
MYC	6	MYC proto-oncogene, bHLH transcription factor	4609	8q24.21	[39, 40, 42, 44]
DLG2	6	Discs large MAGUK scaffold protein 2	1740	11q14.1	[39, 42]
LEPREL1	6	Prolyl 3-hydroxylase 2	55214	3q28	[42, 44]
CASC8^a^	9	Cancer susceptibility 8	727677	8q24.21	[39, 40, 44]
TP63	6	Tumor protein p63	8626	3q28	[39, 40, 42, 44]
ENTPD5	5	Ectonucleoside triphosphate diphosphohydrolase 5	957	14q24.3	[39]
PARD3B	5	Par-3 family cell polarity regulator beta	117583	2q33.3	[41, 42]
PVT1^a^	5	Pvt1 oncogene	5820	8q24.21	[44]
SEMA3D	5	Semaphorin 3D	223117	7q21.11	[42]
ZFAND3	5	Zinc finger AN1-type containing 3	60685	6p21.2	[39, 42]
FOXP2	5	Forkhead box P2	93986	7q31.1	[42, 44]
PAKN	5	Parkin RBR E3 ubiquitin protein ligase	5071	6q26	[42, 44]
TAFA5	5	TAFA chemokine like family member 5	25817	22q13.32	[39, 42, 44]
TPRG1	5	Tumor protein p63 regulated 1	285386	3q28	[39, 42, 43]
ARAP2	4	ArfGAP with RhoGAP domain, ankyrin repeat and PH domain 2	116984	4p14	[39, 42]
BBS9	4	Bardet-Biedl syndrome 9	27242	7p14.3	[39, 42]
CHL1	4	Cell adhesion molecule L1 like	10752	3p26.3	[39, 42]
CNTNAP2	4	Contactin associated protein 2	26047	7q35-q36.1	[39, 42]
AGTR2,	4	Angiotensin II receptor type 2	186	Xq23	[42]
CADM2	4	Cell adhesion molecule 2	253559	3p12.1	[42]
CDH7	4	Cadherin 7	1005	18q22.1	[42]
CPNE8	4	Copine 8	144402	12q12	[42]
DCC	4	DCC netrin 1 receptor	1630	18q21.2	[42]
DUSP6	4	Dual specificity phosphatase 6	1848	12q21.33	[42]
EPHA6	4	EPH receptor A6	285220	3q11.2	[42]
HS3ST4	4	Heparan sulfate-glucosamine 3-sulfotransferase 4	9951	16p12.1	[42]
TEKT4P2^a^	4	Tektin 4 pseudogene 2	100132288	21p11.2	[42]
MSX2	4	Msh homeobox 2	4488	5q35.2	[42]
NEK11	4	NIMA related kinase 11	79,859	3q22.1	[42]
PCDH15	4	Protocadherin related 15	65217	10q21.1	[42]
PLS3	4	Plastin 3	5358	Xq23	[42]
PRDM9	4	PR/SET domain 9	56979	5p14.2	[42]
ZNF33B	4	Zinc finger protein 33B	7582	10q11.21	[42]
IGF1	4	Insulin like growth factor 1	3479	12q23.2	[42, 44]
CNTNAP5	4	Contactin associated protein family member 5	129684	2q14.3	[39, 42]
ERC2	4	ELKS/RAB6-interacting/CAST family member 2	26059	3p14.3	[39, 42]
FGF13	4	Fibroblast growth factor 13	2258	Xq26.3-q27.1	[39, 42]
LINGO2	4	Leucine rich repeat and Ig domain containing 2	158038	9p21.2-p21.1	[39, 42]
RPRD2	4	Regulation of nuclear pre-mrna domain containing 2	23248	1q21.2	[39]
MYO16	4	Myosin XVI	23026	13q33.3	[39, 42]
PTPRN2	4	Protein tyrosine phosphatase receptor type N2	5799	7q36.3	[39, 42]
RELN	4	Reelin	5649	7q22.1	[39, 42]
RGS6	4	Regulator of G protein signaling 6	9628	14q24.2	[39, 42]
SPOCK3	4	SPARC (osteonectin), cwcv and kazal like domains proteoglycan 3	50859	4q32.3	[39, 42]
ZFAT	4	Zinc finger and AT-hook domain containing	57623	8q24.22	[39, 42]
CSMD3	4	CUB and Sushi multiple domains 3	114788	8q23.3	[39, 41, 42]
ERBB4	4	Erb-b2 receptor tyrosine kinase 4	2066	2q34	[39, 41, 42]
CA10	4	Carbonic anhydrase 10	56934	17q21.33-q22	[39, 42]
PDE4D	4	Phosphodiesterase 4D	5144	5q11.2-q12.1	[39, 42]
NLGN1	4	Neuroligin 1	22871	3q26.31	[39, 42]
PROX1	4	Prospero homeobox 1	5629	1q32.3	[40, 42]
ZMAT4	4	Zinc finger matrin-type 4	79698	8p11.21	[39, 42]
TNIK	4	TRAF2 and NCK interacting kinase	23043	3q26.2-q26.31	[39, 40]

^a^Represent the gene type is ncRNA or pseudo, others are protein coding genes

HPV integration normally breaks up the open reading frames of viral E1 and E2 genes which leads to the upregulation of E6 and E7 oncogenes [49]. Genomic instability, HPV integration and gain of telomerase at chromosome 3q26 appear to be strongly associated with genetic events in malignant transformation from CIN to invasive cervical carcinoma. In particular, chromosomal instability may precede genomic integration of oncogenic HPV, while increasing the human telomerase gene copy number occurs after integration as a termination product [50–52]. The integration hotspots are non-random and numerous microRNAs are located in the vicinity of integration hotspots and are influenced by the integrated HPV DNA. Highly homologous stretches of HPV16 viral gene E5 and L2 have been detected at the integration hotspots in independent patients which support themselves as quite important events in the integration process [53]. HPV E6E7 alternative transcripts have shown frequent isoforms in HPV16 or HPV18 positive cervical cancer [54]. Multiple frequent integration sites in human genome have been reported and verified through whole genome sequencing, high-throughput RNA, or chromosome conformation capture (Hi-C) sequencing, whereas the patterns of HPV integration in DNA and RNA samples differ significantly. For instance, DLG2, FHIT, HMGA2, KLF12, KLF5, LRP1B, LEPREL1, LINC00392, POU5F1B, and SEMA3D are DNA hotspots [41, 46]. In addition, CASC8, CASC21, ERBB2, RAD51B, RAP2B, TEX41, TP63, TTC6, MACROD2, MIPOL1, and MYC are hotspot genes in RNA samples [55]. DNA breakpoints are prone to an intron, in contrast, RNA breakpoints are prone to the region of EXON [56]. CCDC106 integration on chromosome 19 has been exhibited in altering local chromosome architecture and structure remodeling [57]. Attentionally, the changes in protein expression levels after HPV integration are inconsistent. FHIT and LRP1B are downregulated, while MYC and HMGA2 are elevated. Moreover, the fusion between HPV and human genome may have occurred by microhomology-mediated DNA repair pathways [46].

In terms of prognostic analysis, HPV16 positive status of the pelvic lymph nodes is a significant predictor of recurrent cervical cancer, while HPV16 integrated form is an unfavorable predictor of overall survival [58, 59]. HPV-DNA integration has been detected with association in carcinogenesis and recurrence free survival [60].HPV integration into the common fragile sites may be associated with distant metastasis [61]. Accurate detection of integration sites will continue with the improvement and combination of multidimensional technologies, such as nanopore sequencing and fluorescent in situ hybridization [62, 63]. Detection methods for viral integration sites are changing rapidly, and we believe that the blueprint for HPV integration will become clearer in the next decades.

## Inferring heterogeneity with human genomics

Genetic intra-tumor heterogeneity acts as a key challenge in tumor evolution and management which affects patients’ outcomes [6, 64, 65]. The fundamental biological mechanisms underlying intra-tumor heterogeneity include genetic drift, selection, heritable variation, and environmental changes [66, 67]. Somatic mutation of FGFR3 has been identified in a large proportion of cervical cancer by Cappellen et al. as early as 1999 [68]. Nevertheless, at least three driver gene alterations are necessary to convert normal cells to malignant cells [69]. Over the past decades, multiple gene expression profiles and novel through-out sequencing studies have focused on capturing intra-tumor heterogeneity over time and space [70, 71]. Whole genome sequencing data among pan-cancer patients (including cervical cancer) has identified 95.1% subclonal expansions of 1705 tumors which verified the importance of intra-tumor heterogeneity [72]. Several scRNA-seq analyses have also been performed to study intra-tumor heterogeneity at the level of individual cells in cervical cancer. We summarize the intra-tumor heterogeneity of cervical cancer from genomic, transcriptomic and epigenetic alterations under different approaches.

### Somatic genomic alterations in cervical cancer

The Catalogue Of Somatic Mutations In Cancer (COSMIC) is the world’s most comprehensive repository of human cancer somatic mutations [73]. Driver hotspots from COSMIC single-base substitution (SBS) mutational signatures are classified into four categories: Deamination, APOBEC, somatic hypermutation, and signature SBS39 [74]. The apolipoprotein B mRNA editing enzyme, catalytic polypeptide-like (APOBEC) which converts cytosine to uracil during RNA editing and retrovirus restriction, has been confirmed in mediating pervasive mutagenesis in human cancers [75]. APOBEC-associated hotspots consist of one to two specific point mutations. In contrast, hotspots associated with somatic hypermutation are characterized by somatic single nucleotide variant (sSNV) clusters in promoter regions, which are clusters of variations in a single nucleotide without any limitations of frequency arisen in somatic cells. APOBEC mutagenesis pattern is associated with 34 common mutational hotspots across multiple cancers and has been identified as the predominant source of mutations in cervical cancers [74, 76]. The high-throughput genotyping platform has been used to interrogate cervical tumors and the consistently high mutation rates of PIK3CA have been confirmed. The APOBEC mutagenesis pattern is associated with nucleotide substitution in the E542K or E545K of PIK3CA, while the non-APOBEC mutagenesis pattern coexists at the same time [44].

The recognized mutated genes are ARID1A, CASP8, EP300, ERBB3, FBXW7, HLA-A, HLA-B, KRAS, MAPK1, NFE2L2, PIK3CA, PTEN, SHKBP1 and TGFBR2 in cervical cancer. It’s worth noting that over 70% of CCs exhibit genomic alterations in PI3K-MAPK and TGFβ signaling pathways [44, 77]. Novel significantly mutated genes have been discovered through deep RNA sequencing approaches and clustering of their mutant allele fraction variants. At least 20% of cervical cancers harbor somatic LKB1 mutations. Approximately 100% of tumors with these mutations harbored single nucleotide substitutions, identifiable monoallelic or biallelic deletions or multiplex ligation probe amplification (MLPA) [78]. Mutational sequencing has identified that 40% of 23 cervical cancer specimens harbored somatic mutations of NOL7, a tumor suppressor gene located on 6p23. Multiple CpG dinucleotides have been detected spanning the first exon or the 5’ untranslated region of NOL7, resulting in its inactivation [79].

There is heterogeneity in gene mutations among different pathological types. PIK3CA mutation rates keep consistent between adenocarcinomas and squamous cell carcinomas. The major mutations in squamous cell carcinomas include EP300, FBXW7, MAPK1, NFE2L2 and EGFR, while KRAS, ELF3, and CBFB in adenocarcinoma [80, 81]. The Cancer Genome Atlas (TCGA) Research Network has identified high frequencies of ARID1A, KRAS, and PTEN mutations in endometrial-like cervical cancers [48]. Mutations in PIK3CA, KRAS, and TP53 have also been detected most commonly in small cell cervical cancer using next generation sequencing [82].

### Differential gene expressions in cervical cancer

To discover transcriptomic intra-tumor heterogeneity, previous studies have investigated differential transcript gene expressions between normal and cervical cancer tissues through microarray technologies [83–88]. At the RNA level, gene expressions determined by the expression profiling microarray are detected by reverse transcription-polymerase chain reaction (RT-PCR). While at the protein level, the expressions of specific proteins are often described in immunohistochemical (IHC) staining. Multiple-gene transcript signature with differential expressions by cDNA microarray could be used for molecular classification between stage IB and IIB and prediction of response to radiotherapy for advanced cervical cancer [85, 86, 89]. Differential expressions of CDKN2A and PTGES have been identified in invasive cervical cancer versus normal keratinocytes through oligonucleotide microarrays and confirmed through immunohistochemical staining [90]. Apoptotic genes BCL2, BCL2l1, and BIRC2 have been identified as upregulated in late-stage cervical cancer compared to early-stage cases [91]. DPP4, EDN3, FGF14, TAC1 and WNT16 have been indicated simultaneously downregulated and hypermethylated in cervical cancer [92]. Message RNA expression levels of RhoB and STMN1 have been validated associated with overall survival in cervical cancer [93]. A positive correlation has been observed between gene expression of HPV E6/E7 oncogenes and UHMK1 [94].

Expression profiling has been replaced gradually by more accurate sequencing techniques and the search for differential expressed genes (DEGs) in tumors continues. Three DEGs, including RDH12, UBD, and SAA1 have been screened with correlation to tumor size, lymphatic metastasis, and depth of cervical invasion in cervical squamous cell carcinoma through RNA sequencing [95]. Upregulated expression of AKT3 in cervical cancer has been related to resistance to cisplatin [96]. Transcriptome sequencing in HPV16 positive cervical cancer tissues has identified 140 DEGs enriched in cell cycle and DNA repair [97].

### Heterogeneity analyzed by single-cell RNA sequencing approaches

Single-cell sequencing is a promising systematic and comprehensive approach to delineating subclone associations and intratumor heterogeneity. Conclusions of single-cell sequencing researches have provided a deeper understanding of specific mechanisms leading to heterogeneity in recent years. The landscape of heterogeneity within 22 cancer cell lines has identified twelve recurrent heterogeneous programs (RHPs) even without the native tumor microenvironment. These RHPs are associated with cell cycle, stress responses, epithelial-mesenchymal transition, and protein metabolism [98]. Focusing on cervical cancer, single-cell RNA sequencing data of 20,938 cells have divided tumor cells into four subpopulations with distinct signature genes and prognoses. Specifically, the cells in the first subpopulation are enriched in immune regulation signaling pathways, such as the ErbB signaling pathway; the cells in the third subpopulation are suggested with high proliferative activity because of their high expression of MKI67, CCNB1 and TOP2A genes. The last two subpopulations are regarded as the original cancer cells and the terminal cancer cells respectively, one with over-expressed stem-related genes SOX2 and ALDHA1 and the other with high expressions of genes enriched in steroid biosynthesis, mismatch repair and peroxisome pathways [99]. Another single-cell RNA sequencing data of 24,371 cells aiming to comprehensively analyze chemotherapy resistant cervical cancer cells have clustered cells into nine subpopulations. Differentially expressed genes enriched in the PI3K/AKT pathway are involved in chemotherapy resistance [100]. The main limitations of microarray and sequencing technologies are detecting variations at the DNA or RNA level rather than the protein level. Validation studies in conjunction with proteomics are essential. Cellular heterogeneity is being characterized in cervical cancer with the advent of single-cell genomics which may provide more accurate information on cancer characteristics, prognostic prediction, and treatment decision selection.

### Epigenetic landscape in cervical cancer

Tumor development and drug resistance are sometimes driven by potential gene amplification and deletion, not only somatic genomic alterations but also copy number amplifications, histone modification, and DNA methylation. A large-scale genomic study, including genomic, transcriptomic, and epigenomic landscapes of 118 Ugandan cervical cancer patients has been performed. DNA methylation, histone marks, and gene expression dysregulation differ between A9 and A7 HPV clades. Clade A7 corresponded to a less differentiated phenotype of cervical cancer and lead to a poorer prognosis. Changes in histone modification are associated with HPV integration [101]. Another comprehensive genomic analysis including whole exome sequencing, copy number and methylation analysis of 228 primary cervical cancers has revealed amplifications in immune checkpoint genes PD-L1 and PD-L2, together with lapatinib associated gene BCAR4 [102]. A C-score model according to the chromosomal-arm-level copy number alterations (CNAs) changes of 1q, 2q, 3p, and 7q has been validated to distinguish ICC from normal tissues with 100% sensitivity and specificity [103].

Deregulation of micro-RNA (miRNA), long non-coding RNA (lncRNA) and circular RNA (circRNA) have also been revealed in cervical cancer patients in recent researches. Specifically, miRNAs are small non-coding RNAs which can regulate gene expression through binding to DNA or mRNA [104]. While lncRNAs are long non-coding RNAs which can regulate gene transcription mediated by interacting with chromatin-modifying complexes and miRNAs [105]. CircRNAs are also small non-coding RNAs playing big parts in post-transcription and participate in genetic expression [106]. A type of endogenous RNA, specifically, competing endogenous RNAs (ceRNAs) have been identified to influent the target genes by miRNA and participate in cancer regulation process ultimately [107]. The ceRNA-miRNA-mRNA regulatory axis is gradually explored in cervical cancer research. Both lncRNAs and circRNAs may function as sponges or ceRNAs of miRNAs to regulate mRNA expression [108]. A recent review summarized the reciprocal regulation role of miRNAs, lncRNAs and circRNAs in CC patients. The miRNAs are divided into “oncogenic” miRNAs (miR-10a, miR-19, miR-21, and miR-146a et al.) and “tumor suppressive” miRNAs (miR-29a, miR-214, miR-218, and miR-372 et al.) [109]. Around 14 lncRNAs have shown to be altered and affected important metabolic pathways such as STAT3, wnt/β-catenin, PI3K/AKT, and Notch signaling in cervical cancer [110]. LncRNA XLOC_006390 can serve as a ceRNA and has been verified reversely regulating the expression of miR-331-3p and miR-338-3p, and facilitating tumorigenesis or metastasis in cervical cancer [111]. CircRNA_VPRBP regulates miR-93-5p/FRMD6 axis which lead to inhibited proliferation, migration and invasion of cervical cancer cells [112]. Furthermore, circRNA hsa_circ_0000515 acts as a miR-326 sponge, has been demonstrated to promote cervical cancer progression through upregulated ELK1 expression [113]. These findings might enumerate the regulatory mechanisms of epigenetics in the development of cervical cancer. However, the complexity interaction among diverse non-coding RNAs shows great heterogeneity, which still needs to be further verified.

## Inferring heterogeneity with therapeutic diversity

**Fig. 5:**
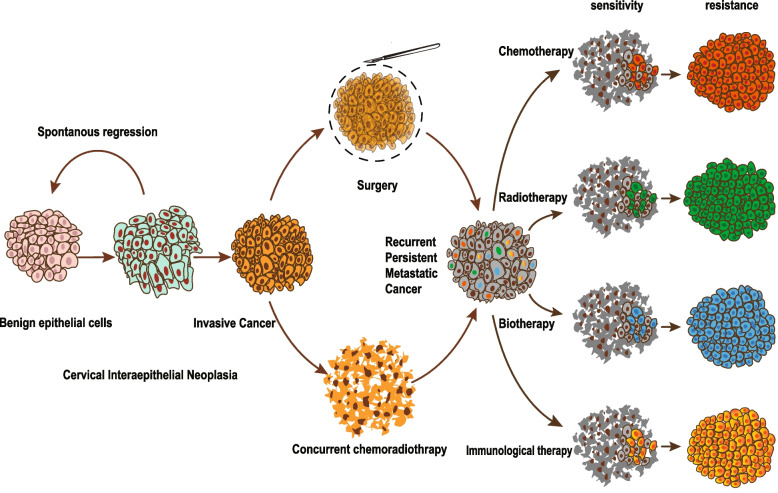
Model of clonal progression of cervical cancer. Normal cervical cells may harbor genomic alterations and HPV integration after HPV infection. Some cells regress to normal spontaneously, while others round into clonally invasive carcinoma cells. Overwhelming majority cancer cells are removed or killed during conventional surgery and chemoradiotherapy. A few dormant or new subclones develop into recurrent, persist or metastatic cancer lesions. Systemic therapies (chemotherapy, radiotherapy, biotherapy and immunological therapy) can induce intrinsic or adapted resistant subclones. Resistant subclones contribute to uncontrolled disease and treatment failure

HPV screening and classic three-step diagnostic criteria have been quite normalized and widely used worldwide in the detection of early-stage cervical cancer. According to clinical guidelines, standard surgical treatment is the first-line recommended with satisfactory effect. Results of a completed randomized controlled phase III trial (NCT00002536) have shown no significant differences in stage IB cervical cancer patients with or without neoadjuvant chemotherapy [114]. For locally advanced cervical cancer patients, adjuvant chemotherapy and radiotherapy can improve patients’ outcomes but with increasing controllable toxicity [115]. Nevertheless, a minority of patients have to face distant metastasis, recurrent or persistent cancer and drug resistance. Combination of multiple-agent chemotherapy and biotherapy (Bevacizumab) may be associated with death reduction and prolonged PFS in recurrent/persistent cervical cancer patients (Table 2) [116]. Tumor heterogeneity is undoubtedly an important factor leading to the reverse therapeutic effects among individuals. Diverse treatment is a double-edged sword because it serves as an external factor to promote persistent adaption and selection in the tumor microenvironment. We suggest that cell subclones are derived in response to different therapeutic stimuli and determine patient outcomes (Fig. 5).

**Table 2:** Completed randomized controlled Phase III trials in cervical cancer

Trial identifier	Brief title	Actual Enrollment	Stage	Arm	Outcomes
NCT00002536	Surgery with or without chemotherapy in treating patients with stage IB cervical cancer	288	IB	Arm I: RHPPL Arm II: NACT + RHPPL	Not statistically significant
NCT00191100	Comparative study of gemcitabine, cisplatin and radiation versus cisplatin and radiation in cancer of the cervix	515	IIB to IVA	Arm I: Gemcitabine + Cisplatin + Brachytherapy Arm II: Cisplatin + Brachytherapy	PFS (HR = 0.68; 95% CI = 0.49–0.95, *p* = 0.0227) and OS (HR = 0.68; 95% CI = 0.49–0.95, *p* = 0.0224) were improved in arm I vs arm II
NCT00803062	Paclitaxel and cisplatin or topotecan with or without bevacizumab in treating patients with stage IVB, recurrent, or persistent cervical cancer	452	IVB, recurrent, or persistent	Arm I: Bevacizumab + Chemotherapy Arm II: Chemotherapy	Median OS was improved in arm I vs arm II (17.0 vs. 13.3 months, HR = 0.71; 98% CI = 0.54–0.95, p = 0.004)
NCT00003945	Comparison of three chemotherapy regimens in treating patients with stage IVB, recurrent, or persistent cervical cancer	294	IVB, recurrent, or persistent	Arm I: Cisplatin + Topotecan Arm II: Cisplatin	Median OS (9.4 vs. 6.5 months, *P* = 0.017) and PFS (4.6 vs. 2.9 months, *P* = 0.014) were improved in arm I vs arm II
NCT00064077	Comparison of four combination chemotherapy regimens using cisplatin in treating patients with stage IVB, recurrent, or persistent cancer of the cervix	513	IVB, recurrent, or persistent	Arm I: Paclitaxel + Cisplatin Arm II: Vinorelbine + Cisplatin Arm III: Gemcitabine + Cisplatin Arm IV: Topotecan + Cisplatin	Best OS (12.87 months) and PFS (5.82 months) in arm I

*RHPPL* Radical hysterectomy and pelvic and para-aortic lymphadenectomy, *NACT* Neoadjuvant chemotherapy, *PFS* Progression free survival, *HR* Hazard ratio, *CI* Confidence interval, *OS* Overall survival

### Heterogeneity in terms of chemotherapy resistance

Cervical cancer chemotherapy can be divided into neoadjuvant chemotherapy (NACT) aiming to shrink the mass to facilitate operation, adjuvant chemotherapy or concurrent chemoradiotherapy (CCRT) as maintenance after surgical treatment or standard treatment for locally advanced patients, and palliative chemotherapy for relieving symptoms, pain or prolonging survival in recurrent or metastatic patients [117]. The majority of these patients will receive more than two combination treatments. Most studies on drug resistance have been limited to in vitro experiments, and few studies have been validated in drug-resistant populations. The molecular mechanism of chemotherapeutic resistance has not been fully understood but could be speculated via blocking DNA damage repair, oxidative stress, autophagy, and apoptosis signaling pathways. Both coding and non-coding RNAs participate in chemo-resistance. Non-coding RNAs, including miRNA, lncRNA, and circRNA, are potential therapeutic targets in cancer treatment development. However, its role in the field of drug resistance of cervical cancer remains to be further explored. Genomic rearrangements may occur by selecting effects from chemoradiotherapy which exhibits genetic intra-tumor heterogeneity in advanced cervical cancers. Platinum-paclitaxel combination chemotherapy is recommended as the first-line chemotherapy drugs in multiple solid cancers and we explain their mechanisms of chemotherapy resistance individually [118].

Cisplatin has been used in most studies of platinum resistance. The mechanisms underlying cisplatin resistance in CC are respectively DNA damage repair increase, apoptosis inactivation, epithelial-mesenchymal transition activation, or DNA methylation alteration [119]. For instance, the upregulated expression of COX-2 has been assessed with neoadjuvant cisplatin-based resistance and unfavorable overall survival in locally advanced CC patients [120]. Cisplatin induces chemotherapy resistance of well-differentiated cell line Caski cells by upregulating Src family kinase and interaction with EphA4 through the reactive oxygen species pathway [121]. Inhibiting endogenous EZH2 expression has shown decreased cell metastasis, reversed cisplatin resistance in HeLa cells, and increased antitumor effects in nude mice. Interfering EZH2 expression has been identified correlated with Dicer overexpressed or regulated H3K27 methylation level, which exhibit antitumor activities by interfering the progression of miRNA transcription, and cell cycle and promote cell apoptosis [122]. MALAT1 and PSAT1 could induce resistance in SiHa cells through PI3K/Akt pathway [123, 124].GAS5 could be regulated by P-STAT3 and affect resistance via miR-21/PDCD4 axis [125]. EDC4 could interact with RPA by alleviating DNA damage in cisplatin-resistant HeLa and SiHa cells [126]. IPO4-CEBPD-PRKDC axis is associated with chemoresistance by inhibiting PRKDC-driven DNA damage repair [127]. In addition, an increasing amount of noncoding RNAs have been confirmed and summarized with association to cisplatin resistance [128, 129]. For example, LncRNA HNF1A-AS1 could affect resistance by regulating miR-34b and promoting TUFT1 expression [130]. LncRNA OTUD6B-AS1 could mediate decreased regulation of miR-206 and increased expression of CCND2 [131]. LncRNA NNT-AS1 could improve chemoresistance via the miR-186/HMGB1 axis [132].

Combination chemotherapy with cisplatin and paclitaxel is a standard treatment in recurrent or advanced cervical cancer with an overall response rate of 29%–67% [133, 134]. Meanwhile, confirmed gain of 3q and loss of 11q chromosomes are early events in cancer progression. Subpopulations with differential responses to chemoradiotherapy may be selected into a single intrinsically resistant subpopulation after five weeks of the therapy [135]. Knockdown of Linc00511 could reduce paclitaxel resistance by regulating Bcl-2, MMP-2, MMP-9, MRP1, and P-GP expressions in HeLa cells [136]. Overexpressed miR-214 under paclitaxel treatment could cause an increase in PARP and a decline in PI-3 kinase/Akt levels [137]. Circular RNA CircMYBL2 could enhance paclitaxel resistance by upregulating EGFR mediated by microRNA-665 in vitro and promoting tumor growth in vivo [138].

### Heterogeneity in terms of radiotherapy resistance

Radiotherapy for cervical cancer is suitable for locally advanced and recurrent patients or other patients who can’t tolerate surgery. The majority of these patients own a worse prognosis due to advanced FIGO stage. The CCRT is the recommended treatment for advanced cervical cancer compared with radiotherapy alone, because CCRT increases patients’ local control rates and improves prognosis [139]. Integrated bioinformatics analysis on RNA sequencing has identified ten potential biomarkers related to radiotherapy resistance in cervical cancer. The results have indicated overexpression in tumor immune process pathways, including cellular defense response, negative regulation of the immunity, T cell and neutrophil activation, regulation of antigen presentation, and peptidyl-tyrosine autophosphorylation [140]. Other biomarkers, such as HIF-1 could enhance hypoxia-induced radio-resistance via targeting NDRG2 [141]. Overexpressed HOTAIR could promote HIF-1a and lead to radio-resistance in mice [142]. CD147 could induce resistance by regulating the percentage of G2/M phase cells and DNA double-strand breaks repair [143]. RhoC-ROCK2 involved DNA repair pathway is necessary for the radio-resistance phenotype in tumor cells [144]. SEPT9 could affect resistance by interacting with the HMGB1-RB axis and mediating miR-375 [145]. Increased expression of HMGB3 correlated with hTERT could predict poor response to radiotherapy, advanced stage and worse outcome [146]. USP21 is overexpressed in radio-resistant patients and could activate the FOXM1/Hippo signaling pathway [147]. Four specific miRNAs (miR-630, miR-1246, miR-1290, and miR-3138) could promote radio-resistance in vitro [148]. MiR-125 could modulate resistance through the downregulation of CDKN1A [149]. LncRNA UCA1 could promote radio-resistance associated glycolysis in SiHa and HeLa cells via HK2/glycolytic pathway [150]. LncRNA SNHG6 could enhance radio-resistant and promote cell growth via STYX/miR-485-3p axis [151]. Tumor radiotherapy has a certain impact on the TME, for instance, the generation of cancer-associated fibroblasts or macrophages [152, 153].

### Heterogeneity in terms of immunotherapy resistance

After failing platinum-based chemotherapy, only about 10% of patients are responsive to additional cytotoxic agents. Immunotherapy of solid tumors is the research hotspot at present aiming to overcome immune suppression in TME and enhance tumor targeted immune attack. The main directions of immunotherapy include immune checkpoint inhibitors, therapeutic antibodies, therapeutic vaccines, cell therapy and small molecule inhibitors. Here we focus on the use of immune checkpoint inhibitors and therapeutic vaccines about the heterogeneity of cervical cancer.

**Fig. 6:**
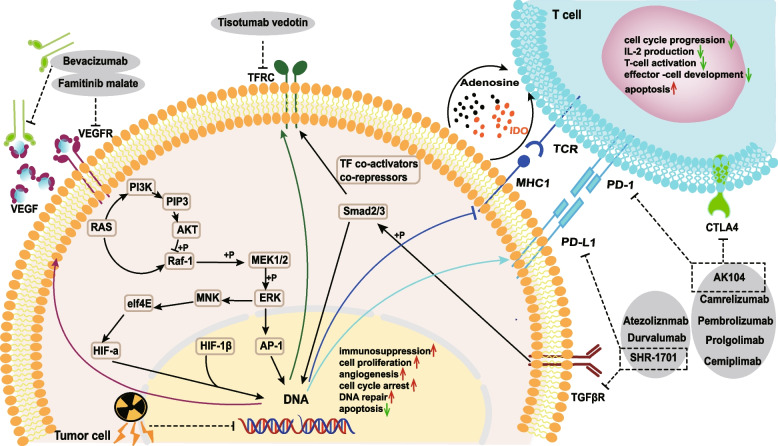
The potential resistance mechanisms and currently on-going phase III clinical trials’ agents in cervical cancer. The drug resistance in tumor cells showed as up-regulation of immunosuppression, cell proliferation, angiogenesis, cell cycle arrest, DNA repair and down-regulation of apoptosis. T cell anti-tumor immunity may be suppressed through down-regulation of cell cycle progression, IL-2 production, T-cell activation, effector-cell development and up-regulation of apoptosis. Agents of clinical trials have focused on novel immune checkpoint inhibitors including PD-1, CTLA4, PD-L1 and TGFβ

Professors James P Allison and Tasuku Honjo won the 2018 Nobel Prize in Physiology or Medicine for discovering CTLA-4 and PD-1 as immune checkpoints and laying the foundation for tumor immunotherapy. The US Food and Drug Administration has already approved pembrolizumab for advanced cervical cancer patients with positive PD-L1. Clinical trials about the efficacy and safety of Pembrolizumab in advanced cervical cancer have been verified. Objective response rate (ORR) refers to the proportion of patients required for the reduction of the tumor to reach the expected value and to continue to the minimum expected time. ORR is commonly to be seen in evaluating the drug response in cancer patients undergoing clinical trials. The ORR of pembrolizumab in these patients has been increased to 14.6% [154]. Results of the phase III clinical trial of KEYNOTE-826 have expanded the indication for combined immunotherapy for persistent, recurrent or metastatic cervical cancer [155]. Results of the phase I/II clinical trial of CheckMate 358 (nivolumab) have shown an ORR of 26.3% with regardless of PD-L1 expression [156]. Three current trials of combining immunotherapy with chemotherapy for cervical cancer involved angiogenesis inhibitors and ICI combination therapy without conclusions (NCT03912415, NCT03635567, and NCT03556839) [157]. We summarized ongoing phase III clinical trials in cervical cancer and illustrated the effect targets for these therapies (Table 3 and Fig. 6). It can be seen from the current ongoing phase III clinical trials in cervical cancer that PD-1 inhibitors include Pembrolizumab, Camrelizumab, Cemiplimab, Prolgolimab (BCD-100), and QL-1604, while PD-L1 inhibitors include Durvalumab and Atezoliznmab. Newly developed dual targeted drugs AK104 (PD-1 and CTLA-4 inhibitors) and SHR-1701 (PD-L1 and TGFβ inhibitors) have already been used in phase III clinical trials. The sensitivity of immunotherapy mainly depends on the heterogeneity of responses between tumor cells, immune-infiltrating cells, and other stroma cells in the TME. With the further development of scientific research, the refinement of immunotherapy indications marks the arrival of the era of precision therapy.

**Table 3:** Ongoing Phase III clinical trials in cervical cancer

Trial identifier	Brief title	Estimated Enrollment	Criteria	Arms and Interventions	Primary outcome measures [Time Frame]	Estimated Study Completion Date
NCT02422563	Neoadjuvant chemotherapy followed by radical hysterectomy (op) versus primary chemo-radiation in cervical cancer FIGO stage IB2 and IIB	534	IB2, IIB	Arm I: NACT + Radical hysterectomy Arm II: CCRT	DFS [5 years]	October 2025
NCT02629718	Neoadjuvant chemotherapy + surgery versus surgery in FIGO IB2 and IIA2 cervical cancer	700	IB2, IIA2	Arm I: NACT + Radical hysterectomy Arm II: Radical hysterectomy	DFS [2 years]	December 2022
NCT01101451	Radiation therapy with or without chemotherapy in patients with stage I-IIA cervical cancer who previously underwent surgery	360	I-IIA	Arm I: EBRT/IMRT Arm II: Cisplatin + EBRT/IMRT	RFS [11 years]	December 2021
NCT04723875	Postoperative adjuvant chemotherapy in early-stage cervical cancer that not meet criteria of adjuvant therapeutic according to NCCN guideline	306	IB1, IB2, IIA1	Arm I: Chemotherapy Arm II: No intervention	DFS [3 years]	January 2026
NCT05277688	Adjuvant concurrent chemoradiotherapy versus radiotherapy in early-stage cervical cancer patients	340	IA2-IIB	Arm I: Cisplatin + IMRT Arm II: IMRT	RFS [5 years]	December 2027
NCT00980954	Chemotherapy and pelvic radiation therapy with or without additional chemotherapy in treating patients with high-risk early-stage cervical cancer after radical hysterectomy	238	IA2-IIA	Arm I: CCRT Arm II: CCRT + Chemotherapy	DFS [4 years]	August 2026
NCT04989647	Intermediate risk cervical cancer: radical surgery ± adjuvant radiotherapy	514	IB1-IIA	Arm I: Surgery only Arm II: Surgery + Radiation Therapy	DFS [3 years]	December 2032
NCT03830866	Study of durvalumab with chemoradiotherapy for women with locally advanced cervical cancer	770	IB2 with positive nodes to IVA (FIGO2009)	Arm I: Durvalumab + CCRT Arm II: Placebo + CCRT	PFS [4.5 years]	June 2023
NCT04138992	A study on the efficacy and safety of bevacizumab in untreated patients with locally advanced cervical cancer	150	I-IIIC	Arm I: Bevacizumab + NACT + CCRT Arm II: Bevacizumab + CCRT Arm III: CCRT	DFS [3 years]	May 2022
NCT02853604	Study of ADXS11-001 in subjects with high risk locally advanced cervical cancer	450	Locally advanced	Arm I: Placebo Arm II: ADXS11-001	DFS [5 years]	October 2024
NCT01566240	Induction chemotherapy plus chemoradiation as first line treatment for locally advanced cervical cancer	500	IB1- IVA with positive lymph nodes	Arm I: CCRT Arm II: Chemotherapy + CCRT	OS [5 years]	May 2026
NCT03534713	Induction chemotherapy followed by standard therapy in cervical cancer with aortic lymph node spread	310	IB1-IVA with positive para-aortic lymph nodes	Arm I: NACT + CCRT Arm II: CCRT	OS [3 years]	December2024
NCT03468010	A trial comparing adjuvant chemotherapy with observation after concurrent chemoradiotherapy of cervical cancer (with pelvic or para-aortic node involvement)	432	IB1-IVA with positive lymph nodes	Arm I: CCRT Arm II: Chemotherapy + CCRT	PFS [3 years]	March 2025
NCT05173272	Induction chemotherapy followed by concurrent chemoradiation in advanced cervical cancer	286	IB3-IIIB	Arm I; NACT + CCRT Arm II: CCRT	PFS [3 years]	February 2028
NCT04974346	Para-aortic prophylactic irradiation for locally advanced cervical cancer	450	IB2-IV with positive pelvic lymph nodes and negative common iliac and paraaortic lymph nodes (FIGO 2009)	Arm I: Para-aortic Prophylactic Irradiation + CCRT Arm II: CCRT	PFS [3 years]	August 2030
NCT05235516	A study of AK104/placebo combined with chemoradiotherapy for the treatment of locally advanced cervical cancer	636	IIIA-IVA	Arm I: AK104 + CCRT Arm II: Placebo + CCRT	PFS [4.5 years]	May 2029
NCT01414608	Cisplatin and radiation therapy with or without carboplatin and paclitaxel in patients with locally advanced cervical cancer	900	IB1 with node positive, IB2, IIA, IIB, IIIB, or IVA (FIGO 2008)	Arm I: CCRT Arm II: CCRT + Chemotherapy	OS [5 years]	July 2022
NCT05189028	Study of neoadjuvant chemotherapy versus definite concurrent chemoradiotherapy for locally advanced bulk cervical cancer	290	IB3, IIA2, IIB-IVA	Arm I: NACT Arm II: CCRT	OS [2 years]	June 2025
NCT04221945	Study of chemoradiotherapy with or without pembrolizumab (MK-3475) for the treatment of locally advanced cervical cancer	980	IB2-IVA with positive nodes (FIGO 2014)	Arm I: CCRT + Pembrolizumab Arm II: CCRT + Placebo	PFS [38 months] OS [46 months]	December 2024
NCT03635567	Efficacy and safety study of first-line treatment with pembrolizumab (MK-3475) plus chemotherapy versus placebo plus chemotherapy in women with persistent, recurrent, or metastatic cervical cancer	600	Persistent, recurrent, metastatic	Arm I: Pembrolizumab + Chemotherapy ± Bevacizumab Arm II: Placebo + Chemotherapy ± Bevacizumab	PFS [2 years] OS [2 years]	November 2022
NCT04906993	Camrelizumab combined with famitinib malate for treatment of recurrent/metastatic cervical cancer	440	Recurrent, metastatic	Arm I: Camrelizumab + Famitinib malate + Chemotherapy Arm II: Chemotherapy	PFS [2 years] OS [2 years]	May 2023
NCT04733820	Clinical efficacy of adjuvant chemotherapy in patients with locally advanced cervical cancer who did not meet the NCCN guidelines for adjuvant treatment after NACT combined with surgery	340	IB3-IIB	Arm I: Chemotherapy Arm II: No intervention	DFS [5 years]	February 2028
NCT05367206	Neoadjuvant chemotherapy followed by chemoradiation versus chemoradiation for stage IIIC cervical cancer patients: a randomized phase III trial	280	IIIC	Arm I: albumin-bound paclitaxel and carboplatin + CCRT Arm II: CCRT	PFS [3 years]	March 2027
NCT03556839	Platinum chemotherapy plus paclitaxel with bevacizumab and atezolizumab in metastatic carcinoma of the cervix	404	IVB, persistent, recurrent	Arm I: Chemotherapy + Bevacizumab Arm II: Atezolizumab + Chemotherapy + Bevacizumab	PFS [48 months] OS [48 months]	December 2023
NCT05179239	A study of SHR-1701 plus platinum-containing chemotherapy with or without BP102 (bevacizumab) as first-line treatment in cervical cancer	572	Persistent, recurrent, metastatic	Arm I: SHR-1701 + Chemotherapy + Bevacizumab Arm II: SHR-1701 + Chemotherapy ± Bevacizumab Arm III: Placebo + Chemotherapy ± Bevacizumab	AEs [21 days] PFS [10 months] OS [26 months]	May 2025
NCT04982237	A study of AK104 plus platinum-containing chemotherapy ± bevacizumab as first-line treatment for persistent, recurrent, or metastatic cervical cancer	440	Persistent, recurrent, metastatic	Arm I: AK104 + Chemotherapy ± Bevacizumab Arm II: Placebo + Chemotherapy ± Bevacizumab	PFS [2 years] OS [2 years]	December 2025
NCT03912415	Efficacy and safety of BCD-100 (anti-PD-1) in combination with platinum-based chemotherapy with and without bevacizumab as first-line treatment of subjects with advanced cervical cancer	316	Recurrent or IVB	Arm I: BCD-100 + Chemotherapy ± Bevacizumab Arm II: Placebo + Chemotherapy ± Bevacizumab	OS [3 years]	December 2024
NCT03257267	Study of cemiplimab in adults with cervical cancer	608	Persistent, recurrent, metastatic	Arm I: Cemiplimab Arm II: Investigator's choice Chemotherapy	OS [40 months]	July 2023
NCT04300647	A study of tiragolumab plus atezolizumab and atezolizumab monotherapy in participants with metastatic and/or recurrent PD-L1-positive cervical cancer	172	Metastatic, recurrent	Arm I: Tiragolumab + Atezolizumab Arm II: Atezolizumab	ORR [3 years]	July 2023
NCT04697628	Tisotumab vedotin vs chemotherapy in recurrent or metastatic cervical cancer	482	Recurrent, metastatic	Arm I: Tisotumab vedotin Arm II: Investigator's choice Chemotherapy	OS [2 years]	May 2024
NCT04864782	QL1604 plus chemotherapy versus chemotherapy in subjects with stage IVb, recurrent, or metastatic cervical cancer	458	Recurrent or IVB	Arm I: QL 1604 + Investigator’s choice Chemotherapy Arm II: Placebo + Investigator’s choice Chemotherapy	AEs [90 days] ORR [6 months] PFS [2 years]	July 2022

*DFS* Disease free survival, *OS* Overall survival, *LACC* Locally advanced cervical cancer, *CCRT* Concurrent chemoradiation therapy, *RFS* Recurrence-free survival, *Chemotherapy* Cisplatin or carboplatin + paclitaxel or docetaxel, *EBRT* Pelvic external-beam radiation therapy, *IMRT* Intensity-modulated radiation therapy, *NACT* Neoadjuvant chem

Novel immune checkpoints, for instance, TIGIT (T cell immune receptor with Ig and ITIM domains) have been utilized combined with anti-PD-1 antibody in recurrent or metastatic cervical cancer (NCT04693234). The application of immune checkpoint inhibitors is limited by the heterogeneity of checkpoint expression on tumor cell surface and immune-activated state in TME. Decreased tumor associated lymphocytes and retained HPV E6/E7 gene expressions may promote treatment resistance during chemoradiation therapy in locally advanced cervical cancer patients [158]. Oncogenic E5, E6, and E7 proteins encoded by HR-HPV, especially HPV16 and 18, are implicated in the PD1/PD-L1 pathway leading to increased PD-L1 expression [159–161]. B cells are activated by radiation combined with PD-1 blockade and could improve overall survival in HPV-associated squamous cell carcinomas patients [162]. LSD1 inhibitor combined with anti-CD47/PD-L1 monoclonal antibodies could more effectively inhibit tumor growth in a subcutaneous xenograft model because of increasing the expressions of CD47 and PD-L1 [163]. Other driver genes (PI3KCA, PI3KCB, DVL3, WWTR1 and ERBB2) in regulating immune response or immune cell infiltration are with application prospect [164]. Three single-nucleotide polymorphisms (SNPs), specifically PAX8, CLPTM1L, and HLA genes, are replicated in cervical cancer patients and are associated with cervical carcinogenesis through disruption in apoptotic and immune response pathways [165, 166].

Therapeutic vaccines have also shown some success in patients with advanced cervical cancer. An alphavirus-based treatment vaccine combined with sunitinib and irradiation could elicit superior antitumor effects [167]. HPV recombinant vaccine prime-boost could enhance CD8^+^ T cell mediated tumor cytotoxicity [168]. PD-1 blockade combined with intra-tumoral therapeutic vaccination could elicit HPV16-associated tumor regression in a murine model [169]. The combined application of cervical cancer therapeutic vaccine and immunotherapy has become the general trend at present [170].

## Inferring heterogeneity with histological diversity

The histological diversity of cervical cancer is also a manifestation of tumor heterogeneity. There were significant differences in treatment sensitivity and prognosis among different histological types. In the previous paragraphs, we have mainly discussed the characteristics of cervical squamous cell carcinoma, while in this section we will focus on the molecular and clinical characteristics of cervical cancer stem cells, cervical adenocarcinoma cells, and cervical neuroendocrine cell subtypes.

### Heterogeneity of cervical cancer stem cell

The clonal evolution model and the cancer stem cell (CSC) model have been used to illustrate intra-tumor heterogeneity. In the first model, stochastic mutations in individual tumor cells form in the tumor microenvironment, the superior sub-clonal cells dominate and proliferate under adaptation and selection [171]. Another model highlights the cellular plasticity and mutational differentiation hierarchy formation generated by CSC clones [172, 173]. We attempt to interpret cervical cancer heterogeneity by describing the cell surface biomarkers, molecular mechanism of stem cell regulation and differences in cytological behavior as follows.

Cervical cancer stem-like cells (CCSC) with an expression pattern of CD44 ( +)CD24(-) surface biomarkers have been isolated from HeLa and SiHa cell lines which present higher capabilities in cell growth, self-renew, chemotherapeutic drug and radiation therapy resistance [174, 175]. Another prolonged Trichostatin A-selected HeLa cell expressing Sox2( +)Oct4( +)Nanog( +) markers display enhanced migration, invasion, and malignancy abilities both in vitro and in vivo, which can also be regulated by STAT3 [176–178]. Ubiquitin B has been confirmed as a key gene in the maintenance of Sox2( +)Oct4( +)Nanog( +) CCSC [177]. Hiwi and Gremlin 1 can be regarded as cervical CSC markers because the increased gene expressions facilitate in vitro tumor sphere formation and in vivo tumorigenicity [179, 180]. The extended phenotype of CCSC has been determined with CK-17, p63 + , All + , CD49f + and higher Aldehyde dehydrogenase activity [181]. Besides, the Wnt/beta-catenin pathway is essential to maintain tumorigenicity by microRNA-135a induced CD133( +) CCSC and CCSC related transcription factor levels promoted by LGR5. Wnt3a stimulation may increase tumor sphere size and self-renew [182, 183]. Cancer is a result of uncontrolled cell growth caused by mutations or epigenetic alterations, while cancer stem cell heterogeneity contributes to the whole process of tumorigenesis, recurrence, metastasis and treatment resistance.

### Heterogeneity of cervical adenocarcinoma

Cervical adenocarcinomas comprise approximately 25% of cervical cancer in the USA with higher histological heterogeneity compared to squamous cell carcinoma [184]. The World Health Organization (WHO) classification and a more innovative International Endocervical Criteria and Classification (IECC) are commonly recognized classification criteria [185, 186]. The traditional WHO 2014 system divides cervical adenocarcinomas into serous, mucinous, endometrioid, clear cell and some other types based on pathological features. The IECC 2018 system attempts to subdivide adenocarcinomas into HPV-associated (HPVA) and non-HPV-associated (NHPVA) categories [187]. NHPVAs, in particular gastric type is significantly associated with age, horizontal extent, invasive depth or lympho-vascular invasion, advanced stage, worse disease-free survival (DFS) or disease-specific survival (DSS). Among the HPVAs, invasive stratified mucin producing carcinoma subtypes have shown worse DFS and DSS [188]. According to the revised WHO classification 2020, 92.7% of HPVAs can be recognized by the presence of luminal mitoses and apoptosis in addition to mucinous adenocarcinomas [189, 190]. Distinct molecular profiles have been demonstrated between SCC and adenocarcinoma as mentioned above, which suggests that more tailored treatment strategies are necessary [81]. Gastric-type cervical adenocarcinoma has been detected with somatic mutations in TP53, KRAS, CDKN2A, and STK11. Prevalent mutations of PIK3CA and PTEN enriched in the PI3K/Akt/mTOR signaling pathway has also been identified [191]. Potentially driven mutations have been identified in BRAF, ERBB2 and ERBB3. Copy-number aberrations (CNAs) are deletions or expansions of chromosomes/genes in somatic cells. Low levels of CNAs without recurrent amplifications or homozygous deletions are also confirmed [192]. Further similarities and differences genetic heterogeneity between HPVA and HPV-positive squamous cell carcinoma remain to be further studied.

### Heterogeneity of other rare histological cervical cancer

Neuroendocrine carcinoma of the cervix (NECC) is a variant of CC with accounts for 1–1.5% [193]. A large meta-analysis with 3538 NECC cases has identified a mean recurrence-free survival of 16 months and overall survival of 40 months [194]. The small cell cervical carcinoma (SCCC) is the most common type of NECC with median overall survival ranging between 10–13 months in advanced SCCC [133]. Adjuvant chemotherapy or chemoradiation is associated with higher five-year survival in 188 SCCC patients [195]. FIGO stage is the unique prognostic factor impacting both overall survival and DFS in a multiple retrospective study with 93 SCCC patients [196]. SCCC is specifically associated with HPV18 infection and its genetic alterations are regulated through PI3K/AKT/mTOR, MAPK, and TP53/BRCA pathways [197]. Driven mutation genes KRAS, PIK3CA, IRS2, SOX2 and homogeneous recombination repair genes are potential therapeutic targets [198].

## Conclusions and future perspectives

HPV-associated cervical cancer is a kind of heterogeneous malignant tumor from many perspectives, and its treatment in the advanced stage is extremely difficult. HPV and drug therapy are two extrinsic factors that are closely related to the heterogeneity of cervical cancer. Meanwhile, an in-depth understanding of tumor heterogeneity is a critical issue in developing precision treatment and screening strategies. Our understanding of the molecular and phenotypic heterogeneity in cervical cancer has improved and benefited from the development of deep sequencing and single cell sequencing technology. Nevertheless, it will take time to get breakthrough results on the heterogeneity of tumor microenvironment and treatment responses in advanced cancer. The integration of genomic, transcriptomic and epigenetic information that captures intra-tumoral heterogeneity will reveal the panoramic view of tumor regulatory mechanisms and will promote breakthroughs in precision medicine.

## Data Availability

Data sharing is not applicable to this article as no datasets were generated or analyzed during the current study.

## Abbreviations

CC: Cervical cancer
CCEMC: Cervical Cancer Elimination Modelling Consortium
CCRT: Concurrent chemotherapy
CCSC: Cervical cancer stem-like cell
ceRNA: Competing endogenous RNA
CIN: Intraepithelial neoplasia
circRNA: Circular RNA
CNA: Copy number alteration
COSMIC: Catalogue Of Somatic Mutations In Cancer
CSC: Cancer stem cell
CTL: Cytotoxic T lymphocyte
DEG: Differential expressed gene
DFS: Disease-free survival
DSS: Disease-specific survival
ECM: Extracellular matrix
FIGO: International Federation of Gynecology and Obstetrics
HPV: Human papillomavirus
HPVA: HPV-associated
HR-HPV: High-risk HPV
ICC: Invasive cervical cancer
IECC: International Endocervical Criteria and Classification
IHC: Immunohistochemical
LMICs: Low-income and middle-income countries
lncRNA: Long non-coding RNA
miRNA: Micro-RNA
MLPA: Multiplex ligation probe amplification
NACT: Neoadjuvant chemotherapy
NECC: Neuroendocrine carcinoma of the cervix
NGS: Next-generation sequencing
NHPVA: Non-HPV-associated
OPC: Oropharyngeal cancer
ORR: Objective response rate
PD-L1: Programmed death-ligand 1
PD-L2: Programmed death-ligand 2
PIM: Post infection microenvironment
RHP: Recurrent heterogeneous program
RT-PCR: Reverse transcription-polymerase chain reaction
SBS: Single-base substitution
SCC: Squamous cell carcinoma
SCCC: Small cell cervical carcinoma
scRNA-seq: Single-cell RNA sequencing
sSNV: Somatic single nucleotide variant
TAM: Tumor-related macrophage
Th1: T-helper 1
TIGIT: T cell immune receptor with Ig and ITIM domains
TME: Tumour microenvironment
WGS: Whole-genome sequencing
WHO: Word Health Organization
